# Superoxide Dismutase 2 (SOD2) in Vascular Calcification: A Focus on Vascular Smooth Muscle Cells, Calcification Pathogenesis, and Therapeutic Strategies

**DOI:** 10.1155/2021/6675548

**Published:** 2021-02-24

**Authors:** You-Tien Tsai, Hsiang-Yuan Yeh, Chia-Ter Chao, Chih-Kang Chiang

**Affiliations:** ^1^Nephrology Division, Department of Internal Medicine, National Taiwan University Hospital BeiHu Branch, Taipei, Taiwan; ^2^School of Big Data Management, Soochow University, Taipei, Taiwan; ^3^Nephrology Division, Department of Internal Medicine, National Taiwan University School of Medicine, Taipei, Taiwan; ^4^Graduate Institute of Toxicology, National Taiwan University School of Medicine, Taipei, Taiwan

## Abstract

Vascular calcification (VC) describes the pathophysiological phenotype of calcium apatite deposition within the vascular wall, leading to vascular stiffening and the loss of compliance. VC is never benign; the presence and severity of VC correlate closely with the risk of myocardial events and cardiovascular mortality in multiple at-risk populations such as patients with diabetes and chronic kidney disease. Mitochondrial dysfunction involving each of vascular wall constituents (endothelia and vascular smooth muscle cells (VSMCs)) aggravates various vascular pathologies, including atherosclerosis and VC. However, few studies address the pathogenic role of mitochondrial dysfunction during the course of VC, and mitochondrial reactive oxygen species (ROS) seem to lie in the pathophysiologic epicenter. Superoxide dismutase 2 (SOD2), through its preferential localization to the mitochondria, stands at the forefront against mitochondrial ROS in VSMCs and thus potentially modifies the probability of VC initiation or progression. In this review, we will provide a literature-based summary regarding the relationship between SOD2 and VC in the context of VSMCs. Apart from the conventional wisdom of attenuating mitochondrial ROS, SOD2 has been found to affect mitophagy and the formation of the autophagosome, suppress JAK/STAT as well as PI_3_K/Akt signaling, and retard vascular senescence, all of which underlie the beneficial influences on VC exerted by SOD2. More importantly, we outline the therapeutic potential of a novel SOD2-targeted strategy for the treatment of VC, including an ever-expanding list of pharmaceuticals and natural compounds. It is expected that VSMC SOD2 will become an important druggable target for treating VC in the future.

## 1. Introduction

Vascular calcification, the ectopic deposition of calcium apatite within the vascular wall, was previously thought to be a degenerative process predominantly involving individuals of advanced age. It is now well established that vascular calcification also affects those with diabetes mellitus (DM) and chronic kidney disease (CKD)/end-stage renal disease (ESRD) at an accelerated speed. The presence of vascular calcification confers detrimental cardiovascular influences through impairing vascular compliance and increasing stiffness, elevating systemic resistance and afterload, leading to cardiac hypertrophy, myocardial remodeling, and a greater risk of heart failure-related hospitalization [1]. Mechanically, the stiffened vessels beget a greater cardiac pulsatile force and rising luminal shear stress, which correlates with endothelial dysfunction and atherogenesis involving coronary and peripheral arteries [2]. In addition, the transmission of the exaggerated pulsatile energy to susceptible end organs potentially contributes to dysregulated perfusion and functional disturbances [3]. Several meta-analyses have shown that aortic and coronary artery calcification is predictive of 2- to 3-fold increase in overall and cardiovascular mortality among at-risk populations [4, 5]. Moreover, patients having vascular calcification are revealed to exhibit a significantly higher risk of developing frailty [6], osteoporosis with fragility fractures [7], and sarcopenia [8] compared to those without. Vascular calcification involving lower limbs is also conducive to peripheral artery occlusion, causing intermittent claudication and compromised wound healing [1]. These findings lend support to the notion that vascular calcification is pathologically important and warrants a greater understanding of its pathophysiology.

## 2. Vital Pathophysiological Pieces of Vascular Calcification: Mitochondrial Dysfunction

The recognition of vascular calcification pathogenesis has evolved rapidly over the past decades, expanding from the passive phenomenon of divalent ion imbalances with medial calcium deposition to the active process of well-orchestrated osteoid-like substance secretion by transdifferentiated vascular medial cells, especially vascular smooth muscle cells (VSMCs) [9]. Drivers of this VSMC phenotypic changes are complex; apart from precipitating factors such as advanced glycation end products (AGEs), protein-bound uremic toxins (indoxyl sulfate and *p*-cresyl sulfate), epigenetic effectors (e.g., microRNAs) [10, 11], and a high-phosphate environment, dysfunctional cellular organelles are also pinpointed as a critical contributor [12]. Lysosomal degradation abnormality can be accompanied by the accumulation of *β*-galactosidase and cellular senescence, while dysregulated endoplasmic reticulum (ER) function and nuclear processing will lead to unfolded protein response and DNA damages, respectively [13]. Most importantly, the perturbation of mitochondrial functioning frequently occurs during the course of vascular diseases including atherosclerosis and vascular calcification and is recognized as a cardinal feature in these disorders.

### 2.1. Mitochondrial Dysfunction: An Overview regarding Vascular Diseases

Mitochondria are widely known as the energy factory of a given cell based on its oxidative phosphorylation capacity. Mitochondrial dysfunction leads to the failure of this organelle to generate adequate ATP to meet cellular demand, culminating in energy failure, compromised cellular viability, and tissue remodeling. The spectrum of mitochondrial dysfunction includes but is not limited to mitochondrial dynamic perturbation, mitochondrial protein synthesis impairment, reduced mitophagy, uprising mitochondrial reactive oxygen species (mtROS), and mitochondrial DNA damages [14]. This phenomenon has been described in multiple degenerative disorders, ranging from malignancy, neurodegenerative disorders, DM, and cardiovascular diseases. Among vascular pathologies, prior studies mostly addressed the role of mitochondrial dysfunction in atherogenesis [15]. Mitochondrial dysfunction in endothelial cells (ECs) and VSMCs aggravates atherosclerosis in multiple ways. Anatomically, ECs in atherosclerotic plaques are shown to contain swollen mitochondria with a circular shape and architectural remodeling with loss of the internal structures [16]. Specifically, there is an ultrastructural reduction of cristae number and a detachment of cristae from the inner membrane, accompanied by membrane breakdown at the cellular periphery [16]. In VSMCs residing in atherosclerotic plaques, their mitochondria are similarly enlarged and vacuolated, exhibiting a degenerative tendency [17].

Mitochondrial dysfunction lies at the core of vascular diseases, especially atherosclerosis. Disrupted mitochondria predispose affected ECs and VSMCs to developing oxidative damages following impaired defensive mechanisms (e.g., turnover through mitophagy), enhancing vascular inflammation and senescence [18]. For example, oxidized low-density lipoprotein (ox-LDL) is found to upregulate caspase-3/9 and trigger EC apoptosis through decreasing mitochondrial membrane potential and enhancing mitochondrial permeability [19]. Mitochondrial fusion and mitophagy are also impaired by ox-LDL by means of Opa-1 downregulation [19]. Plaque stabilization is also adversely influenced by the presence of an impaired autophagy efficiency involving culprit cells [20]. In light of these findings, defective mitochondria should be deemed an integral pathologic player in atherosclerosis.

### 2.2. Mitochondrial Dysfunction in Vascular Calcification: A Less Charted Field

Relatively few studies address the association between mitochondrial dysfunction and vascular calcification. Kim et al. first showed that a natural antioxidant termed *α*-lipoic acid capable of restoring mitochondrial health could effectively ameliorate phosphate-induced VSMC apoptosis through regulating the Gas6-Axl-Akt pathway [21]. Mitochondrial dynamics are also impaired during calcification progression; specimens from calcified human arteries showed an increased expression of dynamin-related protein 1 (Drp1), an integral mediator of mitochondrial fission, while Drp1 downregulation in valvular cells could attenuate their tendency of calcification [22]. Polyphenols such as quercetin are also effective against vascular calcification, potentially through inhibiting Drp1 and subsequent mitochondrial fission [23]. Metformin, a biguanide-type antidiabetic agent, is also shown to retard VSMC calcification through improving mitochondrial DNA damages, restoring mitochondrial membrane potential and energy production, presumably by upregulating AMPK signaling pathways [24]. Mitochondrial biogenesis-related proteins, such as nuclear factor erythroid 2-related factor-1 (Nrf-1) and peroxisome proliferator-activated receptor-*γ* coactivator-1*α* (PGC-1*α*), can also be upregulated by metformin treatment [24]. Although it is still inconclusive whether mitochondrial dysfunction directly participates in the pathogenesis of vascular calcification, the above results indirectly support that mitochondrial dysfunction is closely associated with the process of vascular calcification. Presumably targeting mitochondrial dysfunction may aid in the management of vascular calcification.

### 2.3. Superoxide Dismutase 2 (SOD2) and Mitochondrial ROS

mtROS is an important yet underrecognized pathogenic factor in vascular calcification pathogenesis, and not until a decade ago, mtROS was identified as a vital contributor to uremic vascular calcification [25]. High phosphate-induced VSMC calcification was shown to be accompanied by an increased production of mtROS following mitochondrial membrane potential elevation [25]. The rising mtROS is related to the upregulation of Ca^2+^-sensitive intracellular cysteine protease calpain-1, which leads to higher expressions of alkaline phosphatase and attenuates the expressions of ATP synthases and calcification inhibitors [26]. Higher levels of mtROS frequently ensue mitochondrial dysfunction, lower mitochondrial ATP generation, and aggravate the tendency of VSMC apoptosis as well as calcification in aging mice [27].

SOD2, or manganese SOD, is an important defensive mechanism wielded by cells to eliminate free radicals, especially superoxide [28]. Transcribed from *sod2* and synthesized in cytoplasm, SOD2 is subsequently relocated to the mitochondrial matrix, endowed with the responsibility to scavenge superoxide produced by respiratory chain enzymes and to contain mtROS [29]. This subcellular distribution of antioxidative enzymes is important particularly in vascular cells, since molecules activating ROS-defensive machineries may have differential influences on SOD subtypes. A prior study revealed that curcumin, a strong antioxidant, significantly reduced intracellular ROS levels but did not alter the expressions of SOD2 in VSMCs [30]. Judging from the pathologic importance of mtROS during the course of vascular calcification and the primary action of SOD2 in quenching mtROS, we surmise that SOD2 may potentially play a role in the pathophysiology of vascular calcification of different origins, besides that of SOD1.

In the following sections, we will provide a literature-based summary of the relationship between SOD2 and vascular calcification and the potential implications of a SOD2-targeted strategy for treating vascular calcification. We will put emphasis specifically on VSMCs, a *de facto* cellular component which is heavily involved in medial calcification but garners less research attention.

## 3. SOD2 and Vascular Calcification Involving VSMCs: Old Players with New Actions

### 3.1. Sod2-Knockout (KO) Mice and Vascular Diseases

Much insight has been gleaned from the vascular manifestations in Sod2-KO mice. Homozygous deletion of Sod2 leads to early lethality in rodents [31], and only mice with heterozygous Sod2 deletion can be harnessed for vascular phenotype observation. Heterozygous Sod2 deletion mice exhibited an exquisite susceptibility to oxidative stress and tended to have accelerated atherosclerosis due to prominent endothelial dysfunction [32, 33]. However, the influence of Sod2 deletion on the VSMC phenotype was previously unclear. Zhou et al. found that heterozygous Sod2 deletion mice had VSMCs with increased expressions of collagen and matrix metalloproteinase-2 (MMP-2) and decreased elastin levels, followed pathologically by increasing aortic oxidative stress, vascular remodeling, and rising aortic stiffness [34]. Importantly, deficient SOD2 levels in VSMCs contributed to lower expressions of cell survival-responsive genes including Akt and forkhead box O3a (FoxO3a), especially during chronological aging. These mice were inclined to developing extensive atherosclerosis when exposed to hyperlipidemic stimuli, with prominent plaque instability, necrosis, and hemorrhage [35]. Such phenotype was largely attributable to VSMC apoptosis and the upregulation of calpain and matrix degradation related to higher mtROS levels as compared to their wild-type littermates. Several of these upregulated molecules are also involved in the acceleration of vascular calcification, but none of the above knockout mouse studies addressed the extent of aortic calcification in these animals. It would be tempting to speculate whether germline Sod2 deletion predisposes animals to developing vascular calcification in addition to atherosclerosis, especially when they reach an advanced age.

### 3.2. SOD2 in Vascular Calcification: Effect Duality

The potential role of SOD2 in the pathogenesis of vascular calcification is not recognized until roughly a decade ago. Zhao et al. revealed that mtROS served as a trigger for phosphate-induced VSMC calcification and could be effectively counteracted by SOD mimics or SOD2 upregulation [25]. Although Sod2 expressions were not measured in their experiments, indirect evidence suggested that the substantial mtROS elevation at baseline could result from Sod2 downregulation. The suppression of SOD2 in valvular cells and possibly VSMCs nullifies cellular protection against mtROS-induced DNA damage, leading to significantly higher calcification severities [36]. Apart from this action, SOD2 seems to exhibit other influences on VSMC calcification propensity. Dai and colleagues showed that in cultured VSMCs, the activation of high phosphate-induced autophagy in the forms of autophagosome formation and LC3II upregulation was significantly attenuated by the overexpression of Sod2; the retardation of autophagy in VSMCs paradoxically predisposed these cells to developing calcification [37]. Similar findings were affirmed in CKD mice with aortic calcifications. The observed effect on calcification severity might be modified by the intensity of phosphate-induced apoptosis and the degree to which other autophagy-related genes (e.g., autophagy-related 5 (Atg5)) were altered within the affected VSMCs. Results from the above experiments thus support the duality of influences posed by SOD2 during the process of vascular calcification and the presence of a ROS-independent influence exerted by SOD2 in VSMCs.

Given the perceived importance of SOD2 in vascular calcification and its potential Janus-faced effect on calcification tendency, it would be interesting to examine the expressions of Sod2 in calcified vascular tissues *in vivo*. A prior study disclosed that in a CKD rat model with calcium/phosphate/vitamin D-supplemented diet, calcified aortas harbored increased expressions of inflammatory cytokines (interleukins/tumor necrosis factor (TNF)) and NADPH oxidase and ROS levels but halved expressions of antioxidant enzymes including SOD2 [38]. SOD2 thus more likely assumes an anticalcific role in the vascular calcification pathogenesis. Apart from exerting a host effect, SOD2 also acts against a paracrine-related calcification effect from matrix vesicle (MV) release. Chen et al. in *in vitro* experiments showed that calcified primary VSMCs were able to release MVs capable of influencing their noncalcified neighbors, and recipient cells could respond to this tide of spreading calcification through upregulating Sod2 [39]. In their study, mitochondrial dysfunction or ROS levels were similarly unaltered in recipient cells despite an increase in calcium deposition, echoing the potential existence of a ROS-unrelated influence introduced by SOD2.

During the quenching of mtROS, SOD2 activities generate the byproduct, hydroperoxide (H_2_O_2_), which diffuses out of mitochondria and potentially affects a diverse range of physiological and pathological processes [40]. Cytosolic hydrogen peroxide has been shown to modulate protein thiol, alter their activities, and thereby activate multiple signaling pathways including mitogen-activated protein (MAP) kinases, hypoxia-inducible factor (HIF)-1, and its downstream effectors, p53, Nrf2, nuclear factor-*κ*B (NF-*κ*B), etc. [41], all of which have been implicated in the initiation and progression of vascular calcification.

### 3.3. Effects of SOD2 Other Than ROS in Vascular Calcification: Plausible Candidates

Apart from the traditional belief that SOD2 is mainly responsible for lowering mtROS, SOD2 may have plausible influences on other signaling pathways irrespective of the extent of ROS elevation. VSMCs with Sod2 heterozygous deletion exhibit a constitutive activation of Janus kinase 2 (JAK2)/signal transducer and activator of transcription 3 (STAT3), leading to a greater propensity for continuous proliferation [42]. Sod2 overexpression in a carotid artery balloon injury model attenuated the phenomenon of neointima formation and reduced VSMC migration as well as proliferation through suppressing Akt phosphorylation [43]. SOD2 may also counteract VSMC senescence through its action against ROS and subsequent senescence-associated secretory phenotype emergence [44].

SOD2 may also be a surrogate of ambient oxygen concentration within tissues, and oxygen levels are potential modulators of mineralization tendency [45]. Prior studies suggested that the severity of body-wide hypoxia, in the form of serum HIF-1 levels, was independently associated with the presence and the degree of vascular calcification in diabetic patients [46]. It is proposed that a low oxygen level contributes directly to osteogenic differentiation of osteoblasts and VSMCs, presumably though the induction and perpetuation of proinflammatory stimuli and indirectly through its association with other comorbidities that set the groundwork of vascular calcification [47]. Along the same line, Sod2 expressions may additionally be reflective of the activation of several inflammation-related genes, including nicotinamide phosphoribosyltransferase (NAMPT) and cytokines such as interleukins and TNF-*α* [48], while vascular inflammation serves as a predecessor of subsequent vascular calcification. Downregulation of Sod2 can also be an indicator of the defective mitochondrial respiratory chain complex, indirectly contributing to mitochondrial dysfunction and impaired cell viability [49].

**Figure 1:**
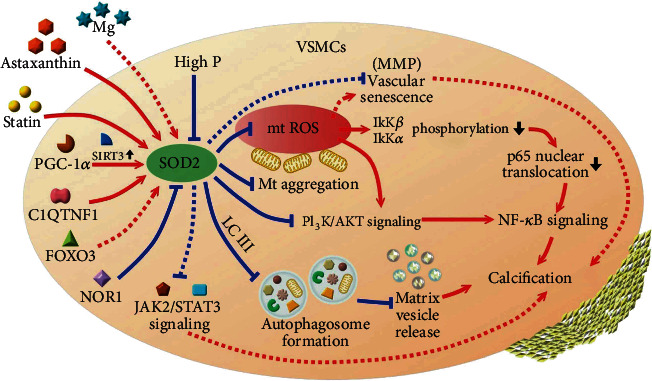
A summary diagram illustrating the regulatory molecules and the effectors of SOD2 in vascular smooth muscle cells demonstrated in the literature. Red arrows indicate activating stimuli, while blue lines indicate inhibitory or antagonistic effects. MMP: matrix metalloproteinase; mt: mitochondria; P: phosphate; ROS: reactive oxygen species; SOD2: superoxide dismutase 2; VSMC: vascular smooth muscle cells.

A brief summary of the above findings regarding the role of VSMC SOD2 in vascular calcification pathogenesis is provided in Figure 1.

## 4. Regulatory Mechanisms for SOD2 Expressions in VSMCs

SOD2 can be transcriptionally regulated in multiple ways. There are several transcriptional activator binding sites located upstream of the promoter of *sod2*, including those of FoxO3a, NF-*κ*B, specific protein 1 (SP1), and activator protein 2 (AP2) [50]. Among these SOD2-regulating molecules, proinflammatory cytokines are the most renowned ones, including interleukin-1, interleukin-6, TNF-*α*, and interferon-*γ* [51]; the activation of SOD2 by cytokines serves as a reflex defensive mechanism during inflammation, ameliorating the damages introduced. ROS is another default modifier of Sod2 expressions. Superoxide radicals have been found to upregulate NF-*κ*B and also Nrf2 levels, thereby inducing Sod2 expressions and enzymatic activities [52]. SIRT3, on the other hand, exerts posttranslational influences on SOD2 enzyme activity and attenuates mtROS [53]. Finally, Sod2 can be epigenetically targeted through CpG island hypermethylation conferred by DNA methyltransferase (DNMT); this methylation-related downregulation of Sod2 also promotes VSMC proliferation involving pulmonary circulation [54].

However, not all regulators of SOD2 found in other cell types have been demonstrated to be in action within VSMCs, and very few studies affirm the relevance of such event to the pathogenesis of vascular calcification. This represents an important research gap waiting to be filled. Contrary to our belief, high glucose exposure is found by some to have minimal influences on Sod2 expressions in VSMCs [55]. We summarized available reports focusing on the upstream regulatory molecules of SOD2 in VSMCs in (Figure 1). Using cultured VSMCs and calcified aortas from CKD rats, Feng et al. disclosed that SIRT3 upregulated Sod2 expressions, attenuated mtROS, and retarded VSMC calcification, while SIRT3 inhibition exhibited the reverse phenotypes [56]. They further discovered that PGC-1*α* was an important endogenous activator of SIRT3 and could effectively defend against vascular calcification through increasing SOD2 levels [56]. Similarly, PGC-1*α* overexpression has been shown to upregulate Sod2 and reduce mtROS production, suppressing VSMC migratory ability both *in vitro* and neointima development *in vivo* [57]. Another smooth muscle cell-specific molecule, SM22, whose function is to bind to actin and anchor the cytoskeleton, also modulates Sod2 expression through deactivating NF-*κ*B. Shen and colleagues identified that primary VSMCs from SM22-KO mice exhibited prominent NADPH oxidase upregulation and NF-*κ*B activation, followed by an increased expression of proinflammatory genes, ROS production, and the feedback upregulation of Sod2 [58]. An adipokine C1q and TNF-related protein 1 (C1QTNF1) is also found to regulate Sod2 expressions in VSMCs; through bioinformatic analyses, Kim et al. showed that VSMCs treated with C1QTNF1 had significant Sod2 upregulation accompanied by activations of interleukin-6 and adhesion molecules [59]. On the other hand, neuron-derived orphan receptor-1 (NOR-1), a ligand-independent transcription factor involved in various growth and inflammatory responses, also plays a role in regulating SOD2 levels. Alonso et al. revealed in VSMCs that NOR-1 induced an upregulation of NADPH oxidase 1 (Nox1) and suppressed Sod2 expressions through lowering p65 nuclear translocation, thereby increasing intracellular ROS levels [60]. Despite the above promising findings, more are needed to facilitate a better understanding of VSMC-specific SOD2 regulatory mechanisms. 1

## 5. SOD2-Targeted Strategies in Vascular Calcification Management

### 5.1. Clinical Evidence of SOD2 Relevance in Vascular Calcification

Judging from the importance of VSMC SOD2 in vascular calcification, it is expected that SOD2 levels differ significantly between clinical specimens from patients with and without vascular calcification. Few studies report on this issue. A group from Italy showed that Sod2 expression levels in the peripheral blood mononuclear cells were significantly higher among patients with coronary artery calcification compared to those without, and this relationship persisted regardless of the patients' DM status [61]. Their findings agree with the experimental results that high glucose levels minimally affect SOD2 levels in VSMCs [55]. Another study reported that plasma obtained from the coronary arteries exhibited significantly greater SOD2 enzymatic activities than those from peripheral blood in patients with coronary atherosclerosis [62]; this suggests that there may be site-specific issues with regard to SOD2 level detection or enzyme activity determination. Andujar-Vera and colleagues examined the proteomic profiles of calcified and noncalcified femoral artery samples from patients with DM and found that Sod2 was an important hub gene that exhibited differential expressions in calcified arterial walls [63]. Based on the above clinical findings, it would be prudent to state that tissue SOD2 levels and circulatory SOD2 activities alter depending on vascular calcification status and severities, and SOD2 likely participates in the pathogenesis of vascular calcification.

### 5.2. Potential Vascular Calcification Treatment Strategies Targeting SOD2

Since SOD2 can be regulated by multiple endogenous molecules and potentially upregulated by exogenous treatments (Figure 1), it would be tempting to repurpose a SOD2-targeted approach for managing vascular calcification. Several reports support the feasibility and potential usefulness of this idea. For instance, statin can be a plausible candidate. Crespo and colleagues demonstrated that VSMCs treated with simvastatin have a downregulation of NOR-1, a negative regulatory molecule of Sod2 [64], and others have indicated that statin can inhibit inflammation-induced vascular calcification *in vitro* [65]. Feresin et al. previously found that polyphenols from berry extracts could trickle up the expressions of Sod2 in treated VSMCs, decrease ROS severity, and ameliorate VSMC senescence, accompanied by the inhibition of Akt, MAPK, and extracellular-regulated protein kinase (ERK) signaling [66]. These effects are expected to improve vascular calcification through the intertwined connections between these pathways and calcification pathogenesis [44, 67]. Magnesium has also been shown to ameliorate oxidative stress and decrease vascular calcification severity through its effect on multiple pathways, including SM22 regulation [68]. Finally, using an *in vitro* model of VSMC calcification in combination with transcriptomic profiling, our research group recently discovered that astaxanthin, a carotenoid derivative with strong antioxidative property, exhibited a prominent anticalcific effect through specifically targeting SOD2 during treatment of calcified VSMCs [69]. Indeed, there are a handful of natural products or extracts exhibiting *in vitro* or *in vivo* effects of augmenting Sod2 expression and activities [70, 71], and testing these SOD2-targeted candidates in VSMCs regarding their anticalcific potential may be a fruitful approach for identifying new pharmaceuticals for treating vascular calcification.

## 6. Conclusion and Future Perspectives

Vascular calcification, although traditionally deemed as an innocent process of vascular aging, has gained tremendous research and clinical attention due to its strong associations with adverse outcomes independent of other confounders. Mitochondrial dysfunction is an emerging pathogenic factor identified during the course of vascular calcification, and mitochondrial ROS lies at the epicenter of this detrimental phenotype. Through counteracting mitochondrial ROS and potentially modulating other signaling pathways, SOD2 acts as a critical gateway within VSMCs blocking the progression of subsequent calcification. Several available pharmaceuticals and natural compounds have been found to upregulate SOD2 specifically in VSMCs, a pivotal pathophysiologic player in vascular calcification, but studies addressing the potential utility of these candidates are scant. More data are needed for uncovering the potential of novel SOD2-targeted strategies for the treatment of vascular calcification.

## Data Availability

No new data are generated from this review.

## Abbreviations

AGE: Advanced glycation end product
AP2: Activator protein 2
Atg5: Autophagy-related 5
C1QTNF1: C1q and TNF-related protein 1
CKD: Chronic kidney disease
DM: Diabetes mellitus
DNMT: DNA methyltransferase
Drp1: Dynamin-related protein 1
EC: Endothelial cell
ER: Endoplasmic reticulum
ERK: Extracellular-regulated protein kinase
ESRD: End-stage renal disease
FOXO3a: Forkhead box O3a
HIF: Hypoxia-inducible factor
JAK2: Janus kinase 2
KO: Knockout
MAP: Mitogen-activated protein
MMP: Matrix metalloproteinase
mtROS: Mitochondrial reactive oxygen species
MV: Matrix vesicle
NAMPT: Nicotinamide phosphoribosyltransferase
NF-*κ*B: Nuclear factor-*κ*B
NOR-1: Neuron-derived orphan receptor-1
Nox: NADPH oxidase
Nrf: Nuclear factor erythroid 2-related factor
ox-LDL: Oxidized low-density lipoprotein
PGC-1*α*: Peroxisome proliferator-activated receptor-*γ* coactivator-1*α*
ROS: Reactive oxygen species
SOD2: Superoxide dismutase 2
SP1: Specific protein 1
STAT3: Signal transducer and activator of transcription 3
TNF: Tumor necrosis factor
VSMC: Vascular smooth muscle cell.
